# Neuregulin 4 attenuates pancreatic β-cell apoptosis induced by lipotoxicity via activating mTOR-mediated autophagy

**DOI:** 10.1080/19382014.2024.2429854

**Published:** 2024-11-14

**Authors:** Biao Zhu, Lei Sun, Junyao Tong, Yan Ding, Yanbo Shan, Mingjuan He, Xiaoyu Tian, Wen Mei, Lisheng Zhao, Ying Wang

**Affiliations:** a Department of Stomatology, Fuxing Hospital, Capital Medical University, Beijing, China; b Department of Stomatology, The Ninth Medical Center,Chinese People’s Liberation Army General Hospital, Beijing, China; c Department of Stomatology, The First Medical Center, Chinese People’s Liberation Army General Hospital, Beijing, China; d Department of Endocrinology, General Hospital of Central Theater Command, Southern Medical University, Wuhan, Hubei Province, China

**Keywords:** AMPK, apoptosis, autophagy, lipotoxicity, mTOR, neuregulin 4 (Nrg4)

## Abstract

Neuregulin 4 (Nrg4) is a brown fat-enriched endocrine factor that ameliorates lipid metabolism disorders. Autophagy is critical for pancreatic β-cell to counteract lipotoxicity-induced apoptosis. This study aimed at exploring whether Nrg4 attenuates lipotoxicity-induced β-cell apoptosis by regulating autophagy. The mouse pancreatic β-cell line MIN6 was cultured in palmitic acid (PA) with or without Nrg4 administration. Apoptosis rate, together with anti-apoptotic and pro-apoptotic protein levels, was investigated. Autophagic flux and autophagy-related protein levels along with related signaling pathways that regulate autophagy were also evaluated. Results showed that Nrg4 decreased PA-induced MIN6 apoptosis, enhanced anti-apoptotic protein B-cell lymphoma 2 (Bcl-2) expression and reduced pro-apoptotic proteins Bcl-2-associated X protein (Bax) and cleaved-caspase 3 expressions. Autophagy levels in MIN6 also decreased with PA treatment and Nrg4 administration reactivated autophagy. Further, Nrg4 administration activated autophagy via the mammalian target of rapamycin (mTOR) signaling pathway. In addition, when the mTOR pathway was stimulated or autophagy was suppressed, the beneficial effects of Nrg4 administration on MIN6 apoptosis were diminished. These results imply that Nrg4 administration attenuates MIN6 apoptosis by promoting mTOR-dependent autophagy and thus may lead to a new therapeutic method for type 2 diabetes mellitus (T2DM).

## 1. Introduction

T2DM, a serious global health problem,^1^ is a metabolic disorder often accompanied by obesity, metabolic syndrome^2^ and elevated free fatty acids (FFA) in plasma.^3^ While fats and glucose are intimately intertwined, increasing FFA in circulating also impairs insulin action, and subsequently elevating blood glucose level. The prevailing hyperglycemic condition induces compensatory synthesis and secretion of insulin, but broken down later, leading to pancreatic β-cell failure.^4–6^ Therefore, lipotoxicity is a major cause of β-cell loss, and methods for the protection of β-cell against lipid overload can be effective for treating T2DM.

Autophagy is an evolutionarily conserved recirculation system, extremely important to maintain cellular and metabolic homeostasis.^7^ Reciprocal communication between autophagy and lipid metabolism in pancreatic β-cell has been proved.^8^ Palmitic acid (PA), a saturated FFA leads to β-cell lines and human pancreatic islets apoptosis due to autophagy inhibition.^9^ Conversely, autophagy activation mitigates islet β-cell dysfunction and apoptosis induced by lipotoxicity.^10^ Therefore, cytoprotective autophagy is critical for β-cell to counteract lipotoxicity-induced apoptosis.

Neuregulin 4 (Nrg4) is a novel adipocytokine that ameliorates lipid metabolism-associated disorders.^11^ In animal studies, Nrg4 has been shown to induce beige fat thermogenesis,^12^ to slow down the transition from hepatic steatosis to steatohepatitis transition,^13^ to alleviate endothelial inflammation and atherosclerosis,^14^ and to promote neurites growth and enhanced innervation to activate thermogenic function.^15^ In addition to these, Nrg4 also ameliorates the severity of diet-induced insulin resistance and hepatic steatosis.^16^ In parallel, several clinical studies have also found that Nrg4 could improve metabolic disturbances. For example, decreased Nrg4 levels were associated with nonalcoholic fatty liver disease in obese children^17^ and were involved in the development of metabolic syndrome in newly diagnosed T2DM patients.^18^ Of note, Nrg4 activates autophagy to alleviate high fat diet-induced hepatic steatosis and PA-induced lipid accumulation in hepatocytes.^7^

Collectively, these findings indicated that lipotoxicity impairs autophagy and consequently causes β-cell apoptosis, while Nrg4 could alleviate lipid metabolism-related disorders and activate autophagy in a high-fat condition. Thus, we hypothesized that Nrg4 could alleviate β-cell apoptosis induced by lipotoxicity via regulating autophagy. We used MIN6, a mouse pancreatic β-cell line to explore the effects of Nrg4 intervention on PA-induced cell apoptosis.

## 2. Materials and methods

### 2.1. Cell culture

MIN6 cells were purchased from Biobuffer Biotech Service Co. (Wuhan, China) and cultured in high glucose (25 mmol/L) Dulbecco modified Eagle medium (DMEM), supplemented with 15% fetal bovine serum (FBS), 100 U/mL penicillin, 100 mg/mL streptomycin, 100 mg/mL L-glutamine, and 5 μL/L β-mercaptoethanol. For cell experiments, MIN6 cells were pretreated with PA (0.4 mm) for 30 min, CQ (10 µM), MHY1485 (10 µM), si Atg7 (50 nM) or 3-MA (2.5 mm), if indicated, was added for another 30 min and then the indicated concentrations of Nrg4 were added. After incubation without changing the culture medium for the indicated time, cells were harvested to be used for assay.

### 2.2. Apoptosis assay

MIN6 apoptosis was determined using flow cytometry after double staining with Annexin V-FITC and propidium iodide (PI), as previously reported.^19^

### 2.3. Secretion studies

MIN6 cells with 5 × 10^5^ per well were used for secretion assays as previously described.^19^ Insulin content was analyzed by ELISA (Millipore) and secreted insulin concentrations at basal (2.8 mm glucose) and stimulated (16.7 mm glucose) levels were normalized to total insulin content.

### 2.4. Monitoring autophagic flux

MIN6 cells were infected 24 hours (hr) before the assay with adenovirus encoding mRFP-GFP-LC3 (10^10^ PFU/ml; Hanbio, Shanghai, China) at 30 multiplicities of infection as previously described.^20^ Autophagosomes were labeled with both red and green signal (mRFP-GFP) and autolysosomes with only red signal (mRFP; GFP proteins quenched in low pH environment in lysosomes). Autophagic flux was monitored using mean fluorescence intensity (MFI) of red/green by flow cytometry.^21^

### 2.5. Western blotting analysis

Proteins obtained from cell extracts were analyzed using western blotting, as previously described^7^. The antibodies used were P62 (#5114, dilution: 1:1000; Cell Signaling Technologies), microtubule‐associated light chain 3B (LC3B) antibody (Ab192890, dilution: 1:2000; Abcam), phosphorylated (p)- adenosine monophosphate‐activated protein kinase (AMPK) antibody (AF3423, dilution:1:1000; Affinity), AMPK (Ab32047, dilution: 1:3000; Abcam), p-mammalian target of rapamycin (mTOR) antibody (AF3310, dilution:1:1000; Affinity), mTOR (AF6308, dilution: 1:1000; Affinity), Bax (Ab32503，dilution: 1:3000; Abcam), Bcl-2 (AF6139，dilution: 1:1000; Affinity), Caspase3 (Ab13847, dilution:1:500; Abcam), Atg5 (Ab108327, dilution:1:3000; Abcam), Atg7 (Ab133528, dilution:1:20000; Abcam) and β-actin (BM0627, dilution: 1:200; Boster, China). The blots obtained were quantified using BandScan 5.0 software.

### 2.6. Immunofluorescence staining

Animal procedures were approved by the Animal Care and Use Committee of General Hospital of Central Theater Command. Eighteen-month-old C57BL/6J mice were maintained on either normal chow (NC, *n* = 30 mice), high-fat diet (HFD, *n* = 30 mice), Nrg4 plus HFD (NRG4, *n* = 30 mice; 100 μg/kg, three times per week) or 3-methyladenine (3-MA) plus NRG4 (NMA, *n* = 10 mice; 30 mg/kg, three times per week, 2 weeks before the end of the study). After 3 months of intervention, pancreas samples were obtained for weighting and immunofluorescence staining as previously described^19^. The antibodies were as follows: insulin (Ab63820, dilution: 1:100; Abcam) and glucagon (#2760, dilution: 1:400; Cell Signaling Technology).

### 2.7. Statistical analysis

Values are expressed as mean ± standard deviation (SD). All statistical analyses were performed using SPSS 26.0 statistics software. Statistical significance was analyzed using one-way ANOVA along with Bonferroni *post hoc* tests when equal variances were assumed or Tamhane’s T2 *post hoc* tests when equal variances were not assumed. Homogeneity of variance was tested using Levene’s test. A *p* value of <0.05 (two-sided) was considered to be statistically significant.

## 3. Results

### 3.1. Nrg4 attenuates pa-induced apoptosis in MIN6 cells

**Figure 1.**
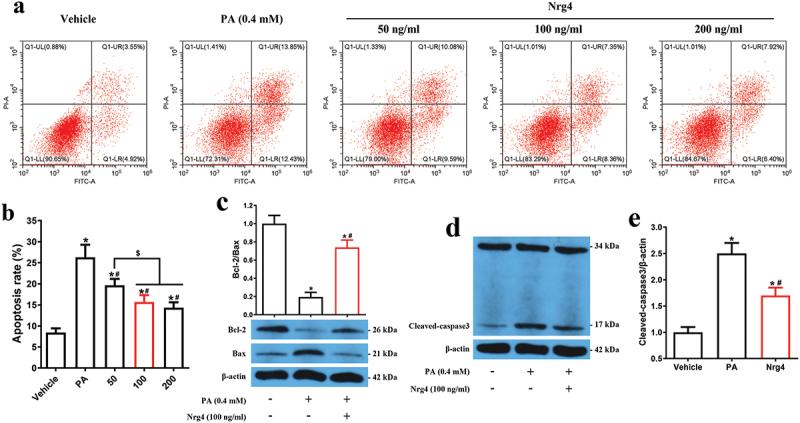
Nrg4 attenuates pa-induced cell apoptosis in MIN6. (a) MIN6 cells were pretreated for 72 hr, and harvested to be used for flow cytometry assay. (b) Quantitative analysis of (a). (c-e) MIN6 cells were pretreated for 72 hr, and harvested to be used for western blotting. (c) Representative immunoblots and densitometric quantification for protein expression of Bcl-2/Bax. (d) Representative immunoblot for the protein expression of cleaved caspase 3. (e) Quantitative analysis of (d). PA, palmitic acid. Data are expressed as mean ± SD, *n* = 3. *, *p* < 0.05 compared with the vehicle group; ^#^, *p* < 0.05 compared with the PA group; ^$^, *p* < 0.05.

It has been demonstrated that prolonged exposure to PA led to pancreatic β-cell apoptosis.^22^ In order to determine the appropriate time for PA to induce MIN6 apoptosis, we first performed a time-response study and found that PA could induce MIN6 apoptosis in a time-dependent manner. Exposure to PA for 72 hr was mostly effective to induce apoptosis (Supplementary Figure S1). Thus, we chose 72 hr as the intervention time to induce apoptosis. Next, we tested the optimal dose for Nrg4 administration. Results showed that Nrg4 attenuated PA-induced MIN6 apoptosis in a dose-dependent but saturated manner (Figure 1a,b). Therefore, we selected 100 ng/ml as the intervention dose in the following experiments. In parallel, analysis of anti-apoptotic protein Bcl-2 and pro-apoptotic proteins Bax and cleaved-caspase 3 revealed that Nrg4 enhanced Bcl-2 expression, which was decreased by PA administration (Figure 1c). On the other hand, Nrg4 suppressed Bax and cleaved-caspase 3 expression, which was increased by PA administration (Figure 1c,e). Together, these results suggested that Nrg4 attenuated PA-induced apoptosis in MIN6 cells.

### 3.2. Nrg4 activates autophagy in pa-treated MIN6 cells

**Figure 2.**
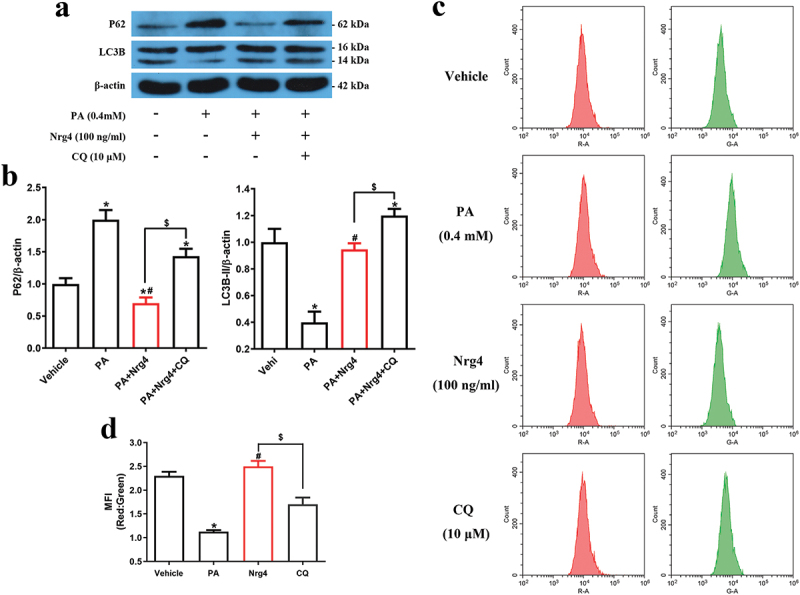
Nrg4 activates autophagy in pa-treated MIN6 cells. (a) MIN6 cells were pretreated for 48 hr, cells were harvested to be used for western blotting. Representative immunoblots for the expression of proteins of P62 and LC3BII (14 kDa). (b) Quantitative analysis of (a). (c) MIN6 cells were pretreated 48 hr and analyzed by flow cytometry. Cells were transfected with adenoviruses encoding mRFP-GFP-LC3 48 hr prior to harvest. (d) Quantitative analysis of (c). PA, palmitic acid. Data are expressed as mean ± SD, *n* = 3. *, *p* < 0.05 compared with the vehicle group; ^#^, *p* < 0.05 compared with the PA group; ^$^, *p* < 0.05.

Autophagy is critical for β-cell to counteract lipotoxicity-induced apoptosis^10^. Therefore, we investigated whether Nrg4 activated autophagy in PA-treated MIN6 cells. To detect the optimal time for Nrg4 administration to activate autophagy, we firstly performed a time-response study. The results showed that exposure to Nrg4 for 48 hr was more effective to increase the LC3B‐II levels than 72 hr (Supplementary Figure S2). To distinguish activated autophagy from the blocked fusion of autophagosomes and lysosomes, leading to the increased LC3B‐II levels, we simultaneously measured P62 protein levels. P62 decrease was also more significant in the 48 hr group (Supplementary Figure S2). Therefore, we chose 48 hr as the intervention time. Secondly, analysis of autophagy-related protein levels revealed that PA significantly decreased LC3B‐II levels but increased P62 protein levels, while Nrg4 administration antagonized the effects of PA. However, when co-treated with autophagy inhibitor chloroquine (CQ), the effects of Nrg4-induced autophagy diminished (Figure 2a,b). Thirdly, considering that autophagy is a dynamic process,^7^ we delivered mRFP-GFP-LC3 double labeled protein into MIN6 to monitor autophagic flux (Figure 2c,d). We found that the MFI of red/green was significantly decreased in the PA group than the vehicle group, indicating reduced autophagy flux. In addition, autophagy flux was significantly increased following Nrg4 administration, while CQ treatment led to a decrease. Collectively, these results implied that Nrg4 intervention could activate autophagy in PA-treated MIN6 cells.

### 3.3. Nrg4 stimulates mTOR-dependent signaling pathway

**Figure 3.**
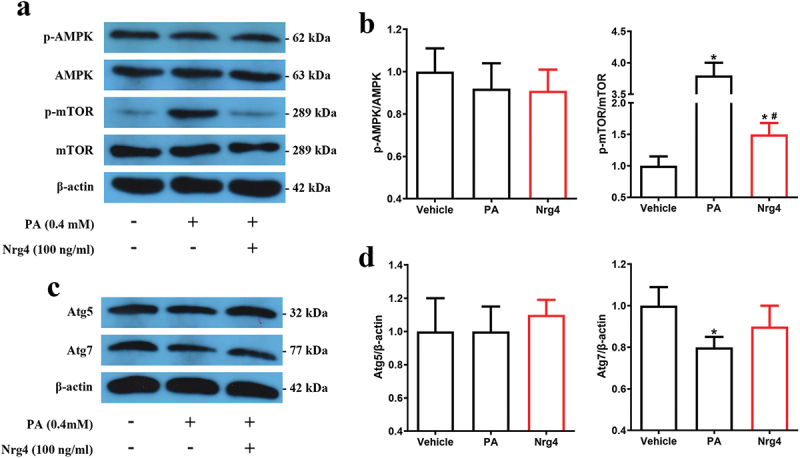
Nrg4 activates the mTOR signaling pathway. MIN6 cells were pretreated for 48 hr, and then harvested to be used for western blotting. (a) Representative immunoblots for phosphorylated (p)-ampk, AMPK, p-mTOR and mTOR. (b) Quantitative analysis of (a). (c) Representative immunoblots for Atg5 and Atg7. (d) Quantitative analysis of (c). PA, palmitic acid. Data are expressed as mean ± SD, *n* = 3. *, *p* < 0.05 compared with the vehicle group; ^#^, *p* < 0.05 compared with the PA group.

Autophagy is mainly regulated by mTOR-dependent signaling pathways^23^ and AMPK serves as a critical upstream regulator that negatively regulates mTOR.^20,24^ Here, we also performed a preliminary time-response experiment to detect the optimal time for the effects of Nrg4 administration on mTOR phosphorylation (Supplementary Figure S3). Results revealed that p-mTOR protein reduction was more obvious in 48 hr than 72 hr. Thus, we selected 48 hr as the intervention time, when mTOR was effectively inhibited. We next explored the p-AMPK and p-mTOR levels (Figure 3a,b) in each group and found that Nrg4 could not activate AMPK, although AMPK tended to be inhibited in the PA group. According to these results, Nrg4 suppressed mTOR, which was activated by PA treatment. Because autophagy-related proteins Atg5 and Atg7 are essential components for autophagosome formation,^25,26^ we determined their protein expression levels (Figure 3c,d). Results showed that Nrg4 supplementation did not affect Atg5 protein expression, but elevated Atg7 protein expression, which was significantly inhibited by PA administration.

### 3.4. mTOR-mediated autophagy is required for Nrg4 attenuated PA induced MIN6 apoptosis

**Figure 4.**
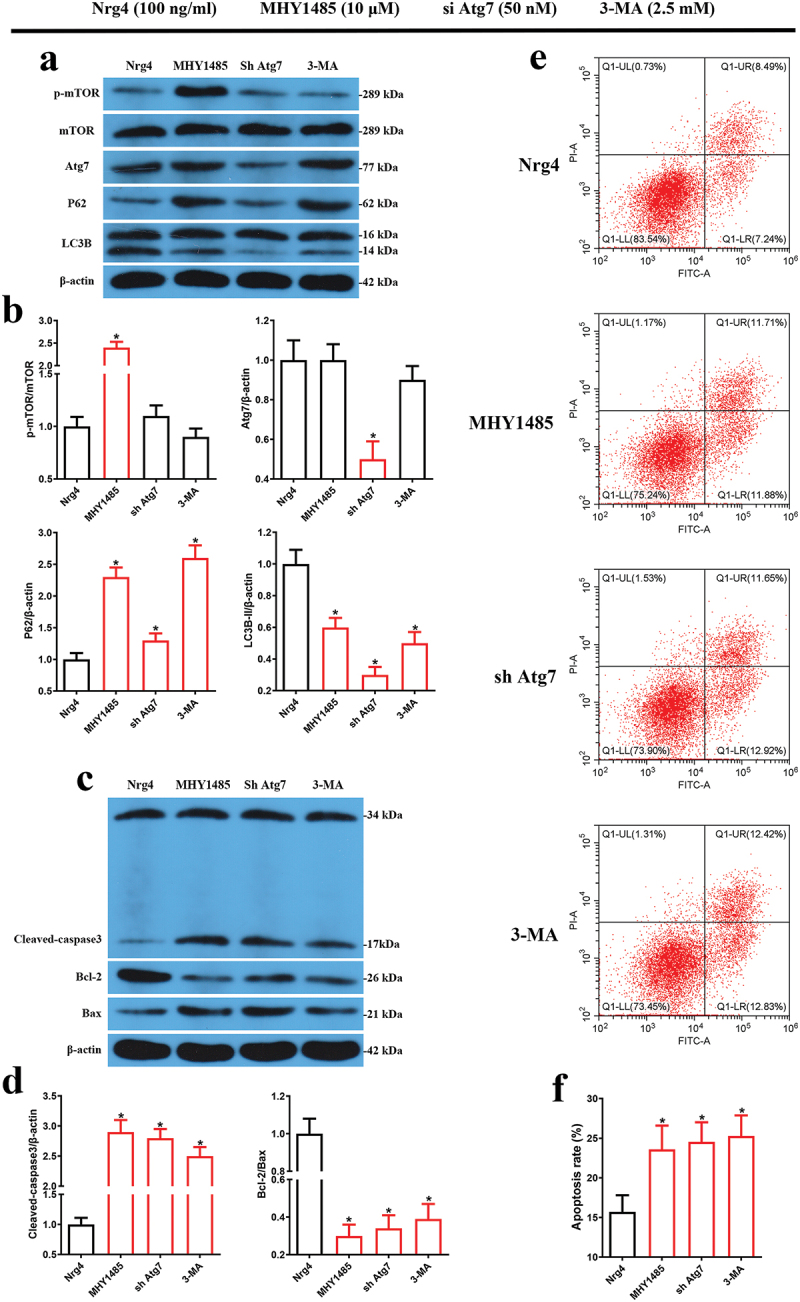
mTOR-mediated autophagy is required for Nrg4 attenuates MIN6 cell apoptosis. MIN6 cells were pretreated with PA, Nrg4 and MHY1485, si Atg7 or 3-MA as indicated for 48 hr, then cells were harvested to be used for western blotting (a-b, c-d) and flow cytometry (c-f), (a) representative immunoblots for p-mTOR, mTOR, P62 and LC3BII (14 kDa); (b) quantitative analysis of (a); (c) representative immunoblots for cleaved caspase3, Bcl-2 and Bax; (d) quantitative analysis of (c). (e) Flow cytometry assay of cell apoptosis; (f) quantitative analysis of (e). PA, palmitic acid. Data are expressed as mean ± SD, *n* = 3. *, *p* < 0.05 compared with the Nrg4 group.

In view of Nrg4 attenuated PA induced apoptosis and activated mTOR-mediated autophagy, we next used MHY1485, an mTOR agonist, to confirm the effects of mTOR-mediated autophagy in Nrg4 attenuated PA induced MIN6 apoptosis. As expected, when mTOR signaling was activated using MHY1485, P62 protein levels were significantly elevated and LC3BII levels were decreased compared to the Nrg4 group (Figure 4a,b), implying suppressed autophagy. Moreover, β-cell specific Atg7 knockout mice exhibited apoptotic β-cell death fed a high fat diet^27^, and our results also showed that Nrg4 could elevate PA-inhibited Atg7 protein expression levels. So, we subsequently used small interfering RNA (siRNA) specific for Atg7 to suppress autophagy (Figure 4a,b). Results showed that P62 levels dramatically elevated while LC3BII levels decreased (Figure 4a,b), also suggesting inhibited autophagy. In addition, 3-MA, a widely-used autophagy inhibitor, could increase P62 levels and reduce LC3BII levels as well (Figure 4a,b). Taken together, these results suggested that MHY1485, si Atg7 and 3-MA could all successfully inhibit Nrg4-induced autophagy.

Next, we determined whether the attenuation of PA induced MIN6 apoptosis by Nrg4 intervention depended on autophagy, we inhibited autophagy using MHY1485, si Atg7 and 3-MA. Then, we found that antiapoptotic protein Bcl-2 all increased, and proapoptotic proteins Bax and cleaved-caspase 3 all decreased, when co-treated with MHY1485, si Atg7 or 3-MA, respectively (Figure 4c,d). Additionally, in constant with the results of apoptosis-related protein analysis, flow cytometry revealed that apoptosis rate obviously elevated after suppressing autophagy using MHY1485, si Atg7 or 3-MA, respectively (Figure 4e,f). Collectively, Nrg4 attenuated PA-induced MIN6 apoptosis, at least partially, depended on mTOR-mediated autophagy.

### 3.5. Nrg4 may attenuate high fat diet induced islet β-cell apoptosis in vivo

Subsequently, we further confirmed the effects of Nrg4 intervention on high fat diet induced islet β-cell apoptosis *in vivo*. Immunofluorescence examination of islet showed that a large insulin‐positive cell core encompassed with an orb of α‐cells in NC and NRG4 mice, while the islet structure of HFD and NMA mice was disorganized (Supplementary Figure S4a). Quantification of β-cell mass showed significant decrease in high fat diet, while Nrg4 intervention could recover β-cell mass. When autophagy was inhibited using 3-MA, β-cells mass sharply decreased (Supplementary Figure S4b). Because the recovered β-cell mass following Nrg4 administration can be a result of increased β-cell proliferation and/or reduced β-cell apoptosis, we next explored the effects of Nrg4 administration on MIN6 proliferation. Proliferation index decreased in the PA group, but Nrg4 could not recover proliferation index to normal levels (Supplementary Figure S4c-d). Thus, we indirectly excluded the possibility of Nrg4 intervention recovered β-cell portion via promoting β-cell proliferation. Collectively, these data indicated that Nrg4 might attenuate high fat diet induced islet β-cell apoptosis *in vivo*.

To further corroborate the role of Nrg4 in islet ß-cell function, we explored the effects of Nrg4 on insulin content and GISS in MIN6 cells. Results showed Nrg4 administration stimulated increase both in insulin biosynthesis (Supplementary Figure S5a) and secretion (Supplementary Figure S5b).

## 4. Discussion

**Figure 5.**
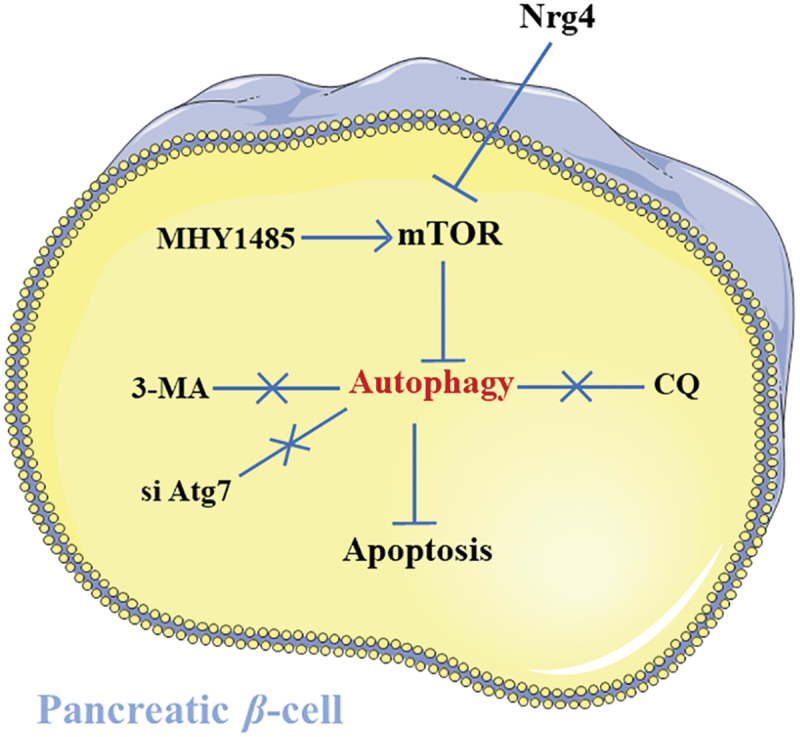
Schematic diagram of Nrg4 intervention. Nrg4 administration inhibits mTOR in β-cell, which sequentially activates autophagy, and then attenuates lipotoxicity-induced β-cell apoptosis. MHY1485, an mTOR agonist, which inhibits autophagy by activating mTOR. While, si Atg7, 3-MA and CQ could directly suppressed autophagy, respectively.

The results of our study elucidated, for the first time, the effects of Nrg4 intervention on the apoptosis of clonal pancreatic β-cells MIN6. The major findings are: (1) Nrg4 administration alleviated PA-induced MIN6 apoptosis; (2) Nrg4 alleviated apoptosis via mTOR-dependent autophagy (Figure 5).

Pancreatic β-cell apoptosis leads to dramatically diminished cell mass, contributing to the onset and progression of diabetes.^28^ β-cells are sensitive to various kinds of stress because of the low expression of critical factors for cell survival.^29^ In addition, lipotoxicity is a major metabolic stress, inducing β-cells apoptosis and obesity-linked diabetes.^10,30^ In this study, we showed that PA promoted MIN6 apoptosis, elevated pro-apoptotic protein expression and reduced anti-apoptotic protein expression. Nrg4, an epidermal growth factor-like extracellular ligand family member, stimulates v-erb-b2 avian erythroblastic leukemia viral oncogene homolog (ErbB) receptor to initiate intracellular effects.^31^ Nrg4, which is highly expressed in the pancreas, regulates lineage determination of developing pancreatic islet cells through ErbB signaling.^32^ It has also been proved that exogenous Nrg4 exerted protective effects on lipid metabolism disorders.^7^ Our study showed that exogenous Nrg4 could counteract the effects of PA’s β-cell toxicity, attenuate apoptosis and improve apoptosis-associated protein expression.

We also explored the underlying mechanisms of Nrg4-attenuated PA-induced apoptosis. We focused on autophagy, which effectively inhibits apoptosis under a variety of stress and diseased states.^33^ Previous studies have shown that autophagy inhibition was the underlying mechanism of lipotoxicity-induced islet β-cell dysfunction and apoptosis.^10,34^ In this study, we considered the activated autophagy as an underlying mechanism for the attenuation of PA-induced β-cell apoptosis by Nrg4 administration based on the following results: 1) autophagy levels of MIN6 decreased in PA treatment, while Nrg4 administration attenuated these decreases; 2) the decreased MIN6 apoptosis caused by Nrg4 administration was spared when suppressed autophagy using 3-MA or si Atg7; 3) autophagy inhibition in NMA mice reduced islet β-cell portion, on the premise that Nrg4 did not affect β-cell proliferation. These results indicated that autophagy, at least partially, mediates the attenuation of β-cell apoptosis through Nrg4 intervention. In addition, widely used anti-diabetic drugs as metformin^35^ and rosiglitazone^36^ also activate autophagy to alleviate PA overload stress.

However, some earlier studies suggested that FFA stimulated autophagy in pancreatic β-cells,^37,38^ and the induced autophagy was considered as an adaptive response to PA treatment. Different pancreatic β-cell type, and exposure time and dosage of PA treatment might contribute to this discrepancy. In addition, although in consistent with our result that FFA inhibited autophagy in pancreatic β-cells, some evidence^9,39^ implied that FFA inhibited autophagy turnover, as confirmed by the significant increase of LC3BII, an early phase marker of autophagy. Unlike our results showed that FFA suppressed autophagic induction, as evidenced by decreased LC3BII levels. One possible explanation for this discrepancy is that, LC3BII is intermediate structure in a dynamic autophagy progress.^40,41^ Thus, the observed LC3BII protein levels depend on the specific observation time point. In other words, exposure time of FFA is different.

Next, we explored probable signaling pathways that may regulate autophagy induced by Nrg4 administration. It has been established that mTOR is a key modulator of autophagy,^42^ and that AMPK, a conserved sensor of energy status, negatively regulates mTOR.^43^ Our results showed that Nrg4 administration suppressed mTOR, and thereby activated autophagy. Furthermore, mTOR-dependent autophagy was necessary for reducing MIN6 apoptosis by Nrg4 administration. However, our data did not reveal any change in p-AMPK expression, thus excluding the possibility of Nrg4 administration activated autophagy through AMPK signaling. Together, these results indicated that the mTOR signaling is most likely the major pathway for autophagy activation through Nrg4 intervention.

Our study has some limitations. First, although our work supported the notion that autophagy reactivation was an underlying reason for the attenuation of β-cell apoptosis through Nrg4 intervention, we could not rule out other mechanisms of action. Furthermore, the data linking autophagy restoration with attenuated β-cell apoptosis were not totally clear. It was reported that endoplasmic reticulum (ER) stress is an essential mechanism for β-cell apoptosis in chronic saturated FFA exposure;^30,44^ therefore, further evidence is needed to clarify whether autophagy reactivation rescues β-cell from apoptosis via alleviating endoplasmic reticulum stress in Nrg4 administration. Second, we stained pancreatic sections for Ki67/insulin positive cells in HFD aged mice, but only few β-cells were Ki67 positive, we can’t conclude the effects of Nrg4 administration on β-cell proliferation in vivo with so less Ki67+ β-cells in pathological sections. So, we have to explore the effects of Nrg4 administration on β-cell proliferation in vitro for second best. But, MIN6 cell line data may not recapitulate HFD aged mouse in vivo data.

## 5. Conclusions

Collectively, this study provides new insights into the attenuation of β-cell apoptosis through the administration of Nrg4, a potent autophagy activator. Autophagy modulation through Nrg4 intervention may represent a promising therapeutic strategy for the management of T2DM.

## Supplementary Material

The clean version Supplementary information R2.docx

## Abbreviations

Nrg4: neuregulin 4
T2DM: type 2 diabetes mellitus
FFA: free fatty acids
PA: palmitic acid
Bcl-2: B-cell lymphoma 2
Bax: Bcl-2-associated X protein
3-MA: 3-methyladenine
DMEM: Dulbecco modified Eagle medium
PI: propidium iodide
FBS: fetal bovine serum
hr: hours
MFI: fluorescence intensity
LC3B: microtubule‐associated light chain 3B
AMPK: adenosine monophosphate‐activated protein kinase
mTOR: mammalian target of rapamycin
NC: normal chow
HFD: high-fat diet
CQ: chloroquine
siRNA: small interfering RNA
SD: standard deviation
GISS: glucose stimulated insulin secretion
ER: endoplasmic reticulum
