# Supplementary material for: Neuregulin 4 attenuates pancreatic β-cell apoptosis induced by lipotoxicity via activating mTOR-mediated autophagy
Source: Islets. 2024 Nov 14;16(1):2429854. doi: 10.1080/19382014.2024.2429854 (PMC11572226; doi:10.1080/19382014.2024.2429854)
Supplement: The clean version Supplementary information R2.docx [file KISL_A_2429854_SM5890.docx]

**Neuregulin 4 attenuates pancreatic β-cell** **apoptosis induced by lipotoxicity via activating** **mTOR-mediated** **autophagy**

Biao Zhu ^1,^ **^†^**, Lei Sun ^2,^**^†^**, Junyao Tong ^3^, Yan Ding ^4^, Yanbo Shan ^3^, Mingjuan He ^4^, Xiaoyu Tian ^3^, Wen Mei^4^, Lisheng Zhao ^3,*^, Ying Wang ^3,*^

1. Department of Stomatology, Fuxing Hospital, Capital Medical University, Fuxingmen Wai Street A 20, Beijing 100038, China.

2. Department of Stomatology, The Ninth Medical Center, Chinese People's Liberation Army General Hospital, Anxiang Bei Road 9, Beijing 100101, China.

3. Department of Stomatology, The First Medical Center, Chinese People's Liberation Army General Hospital, Fuxing Road 28, Beijing 100853, China.

4. Department of Endocrinology, General Hospital of Central Theater Command, Southern Medical University, Wuluo Road 627, Wuhan 430070, Hubei Province, China.

**Supplementary Figure Legends**

**Supplementary Figure 1.** **Time-dependent response of MIN6 apoptosis induced by PA.** (**a**) MIN6 cells were pretreated with or without PA (0.4 mM) for the indicated time, then harvested to be used for flow cytometry assay. (**b**) Quantitative analysis of (**a**). Data are expressed as mean ± SD, *n*=3. PA, palmitic acid. *, *P*<0.05.

**Supplementary Figure 2.** **Time-dependent autophagy-related protein expression of Nrg4 administration.** MIN6 cells were pretreated with PA (0.4 mM) and Nrg4 for the indicated time, then cells were harvested to be used for western blotting. LC3BII, 14 kDa. Data are expressed as mean ± SD, *n*=3. PA, palmitic acid. *, *P*<0.05 compared with the 24 hr group; **^#^**, *P*<0.05 compared with the 48 hr group.

**Supplementary Figure 3. Time-dependent p-mTOR expression of Nrg4 administration.** MIN6 cells were pretreated with PA (0.4 mM) and Nrg4 for the indicated time, then cells were harvested to be used for western blotting. Data are expressed as mean ± SD, *n*=3. PA, palmitic acid. *, *P*<0.05 compared with the 24 hr group; **^#^**, *P*<0.05 compared with the 48 hr group.

**Supplementary Figure 4. Nrg4 may attenuate high-fat diet-induced islet β-cell apoptosis *in vivo*.** (**a**) Representative images of pancreatic sections double-stained for β-cell (green) and α-cell (red), the nuclei were stained with DAPI (blue). Scale bar, 20 µm. (**b**) β-cell mass, *n*=4 mice. (**c**) MIN6 cells were pretreated for 72 hr, cells were harvested to be used for flow cytometric analysis of cell cycles. (**d**) Quantitative analysis of (**c**), proliferation index= (S+G2/M)÷ (G0/G1+S+G2/M), *n*=3. NC, normal chow; HFD, high fat diet; NRG4, Nrg4 plus HFD; NMA, 3-MA plus NRG4; PA, palmitic acid. Data are expressed as mean ± SD. *, *P*<0.05 compared with NC or Vehicle group; **^#^**, *P*<0.05 compared with the HFD group; ^$^, *P*<0.05 compared with the NRG4 group.

**Supplementary Figure 5. Nrg4 attenuates PA-induced islet β-cell disfunction.** MIN6 cells were treated with PA (0.4 mM) for 72 hr with or without the preincubation of Nrg4 (100 ng/ml) for 24 hr. (**a**) Insulin content, (**b**) GSIS analysis, black bars = 2.8 mmol/L glucose, blue bars = 16.7 mmol/L glucose.
